# Purification and characterization of cysteine protease from miswak *Salvadora persica*

**DOI:** 10.1186/s12858-018-0100-1

**Published:** 2018-12-03

**Authors:** Wesam H. Abdulaal

**Affiliations:** Biochemistry Department, Faculty of Science, King Abdulaziz University, Jeddah, Kingdom of Saudi Arabia

**Keywords:** Miswak, *Salvadora persica*, Cysteine protease, Purification, Characterization

## Abstract

**Background:**

Generally, proteases in medicinal plants had different therapeutic effects such as anti-inflammatory effect; modulate the immune response and inhibitory effect toward tumor growth. In this study, protease was purified and characterized from miswak roots, as medicinal plant and natural toothbrush.

**Results:**

Physical and chemical characterization of cysteine protease P1 were studied such as pH optimum (6.5), optimum temperature (50 °C), thermal stability (50 °C) and Km (3.3 mg azocasein/ml). The enzyme digested some proteins in the order of caseine > haemoglobin > egg albumin >gelatin > bovine serum albumin. Hg^2+^ had strong inhibitory effect on enzyme activity compared with other metal ions. Kinetic of inhibition for determination the type of protease was studied. Iodoactamide and *p*-Hydroximercuribenzaoic acid (*p*-HMB) caused strong inhibitory effect on enzyme activity indicating the enzyme is cysteine protease.

**Conclusions:**

The biochemical characterization of this enzyme will be display the suitable conditions for using of this enzyme in toothpaste in the future and the enzyme may be used in other applications.

## Background

Proteases has been characterized from plants such as pea roots [1], ginger [2], *Euphorbia microsciadia* [3] and sweet potato [4]. Plant Proteases had medicinal properties such as bromelain proteases which treated anticancer and osteoarthritis [5–8]. Bromelain has also been effective in the treatment of cardiovascular diseases, where it inhibited the aggregation of blood platelet and minimized the risk of arterial thrombosis [9]. In human intestine, bromelain was absorbed without its degrading and losing its biological activity [10, 11]. Other proteases from malian medicinal plants treated schistosomiasis [12]. Papaya proteases had pharmaceutical applications such as antitumorals, anti-inflammatory, wound healing and digestive disorder [13]. The partially purified protease from *B. subtilis* substantially dehaired cow skin [14].

Chemical compositions of miswak (*Salvadora persica*), natural toothbrush, included salvadoside and salvadoraside [15–17]. Silica removed stains from tooth surfaces [17, 18]. Miswak had several biological activities such as oral hygiene, antibacterial and antifungal [17–21]. Previously, we published article on purification and characterization of α-amylase from miswak [22]. In the present study, proteases from miswak has been purified and characterized.

## Methods

### Miswak

Miswak root was purchased from local market of Jeddah, Saudi Arabia.

### Measurement of protease used azocasein

Proteolytic activity was measured by Dominguez and Cejudo [23]. One unit of protease was defined as μg azocasein hydrolyzed/ml/h under standard assay conditions.

### Ninhydrin assay

α-Amino nitrogen was determined by Moore [24] for the substrates gelatin, casein, egg albumin, bovine serum albumin and hemoglobin.

### Measurement of protein

Protein was measured by Bradford [25].

### Purification procedure of miswak protease

Protease was purified from miswak root by using ion exchange and gel filtration chromatography techniques. By ion exchange, DEAE-Sepharose column (10 × 1.6 cm. i.d.) was used and the elution buffer was Tris-HCl pH 7.2 in presence of gradient of NaCl ranged from 0.0 to 0.3 M. The same buffer was used for gel filtration (Sephacryl S-200 column, 90 × 1.6 cm. i.d.) in absence of NaCl.

### Molecular mass

The molecular mass of purified protease was determined by two techniques, gel filtration and sodium dodecyl sulphate (SDS-PAGE) [26].

### Characterization

Physical and chemical characterization of protease with respect to pH optimum (pH 4–9), optimum temperature (30–80 °C), thermal stability (30–80 °C), substrate specificity (caseine, hemoglobin, egg albumin, gelatin and bovine serum albumin) and effect of metal ions (Ca^2+^, Ni^2+^, Co^2+^, Pb^2+^, Hg^2+^, Cu^2+^ and Zn^2+^) were studied. Kinetics of inhibition (phenylmethylsulfonyl fluoride (PMSF), 1,10 phenanthroline, ethylenediaminetetraacetic acid (EDTA), *p*-HMB and iodoacetamide) for determination the types of protease were carried out.

### Determination of km

The km values were determined from Lineweaver–Burk plots by using different concentrations of azocasein ranged from 1.5–4.5 mg/ml [27].

## Results

**Fig. 1:**
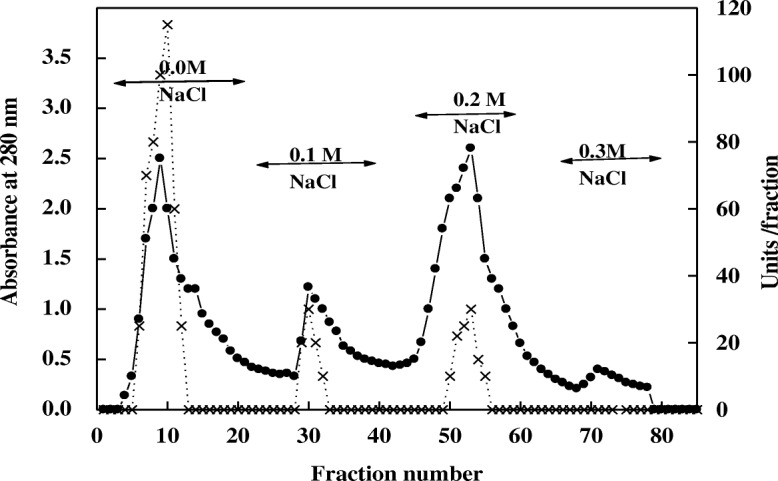
Chromatography of miswak protease on DEAE-Sepharose column. (•^___^•) Absorbant at 280 nm, (x ^___^ x) units/fraction

**Fig. 2:**
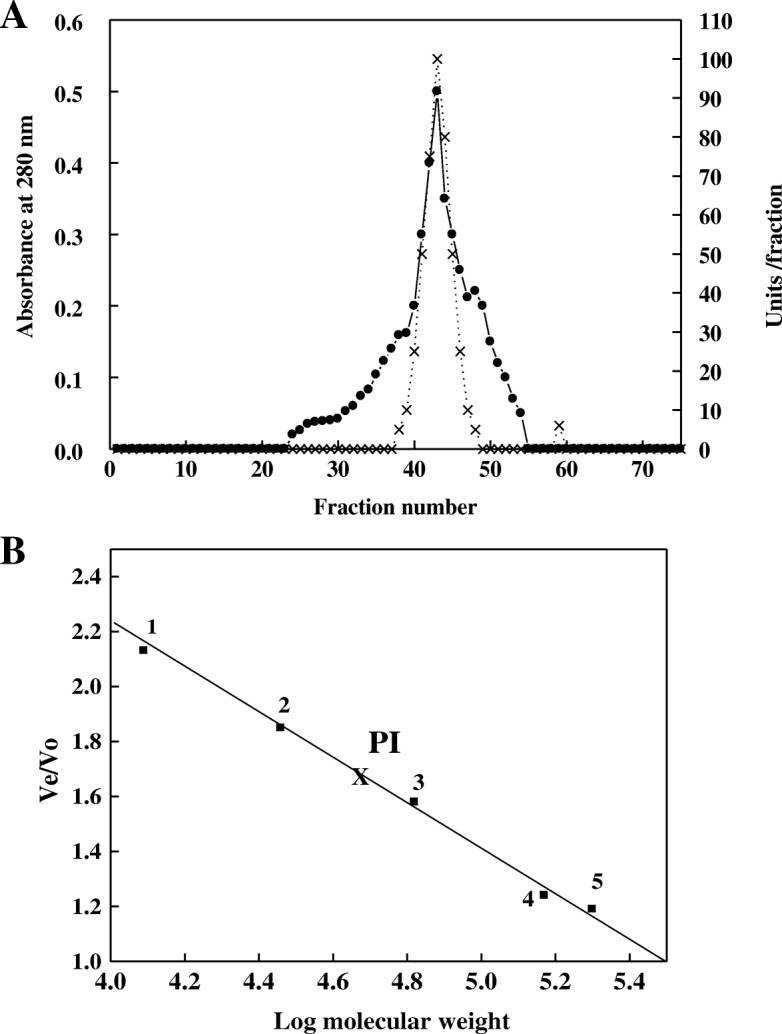
**a** Chromatography of miswak protease P1 DEAE-Sepharose fraction on Sephacryl S-200 column. (•^___^•) Absorbant at 280 nm, (x ^___^ x) units/fraction. **b** Molecular weight value for miswak protease P1 was calculated from calibration curve of Sephacryl S-200 column. Standard proteins from 1 to 5 were 1) Cytochrome C (12,400 Da); 2) Carbonic anhydrase (29,000 Da); 3)Bovine albumin (66,000 Da); 4)Alcohol dehydrogenase (150,000 Da); 5) β-Amylase (200,000 Da). Void volume was determined with Dextran blue (2000, 000 Da)

**Fig. 3:**
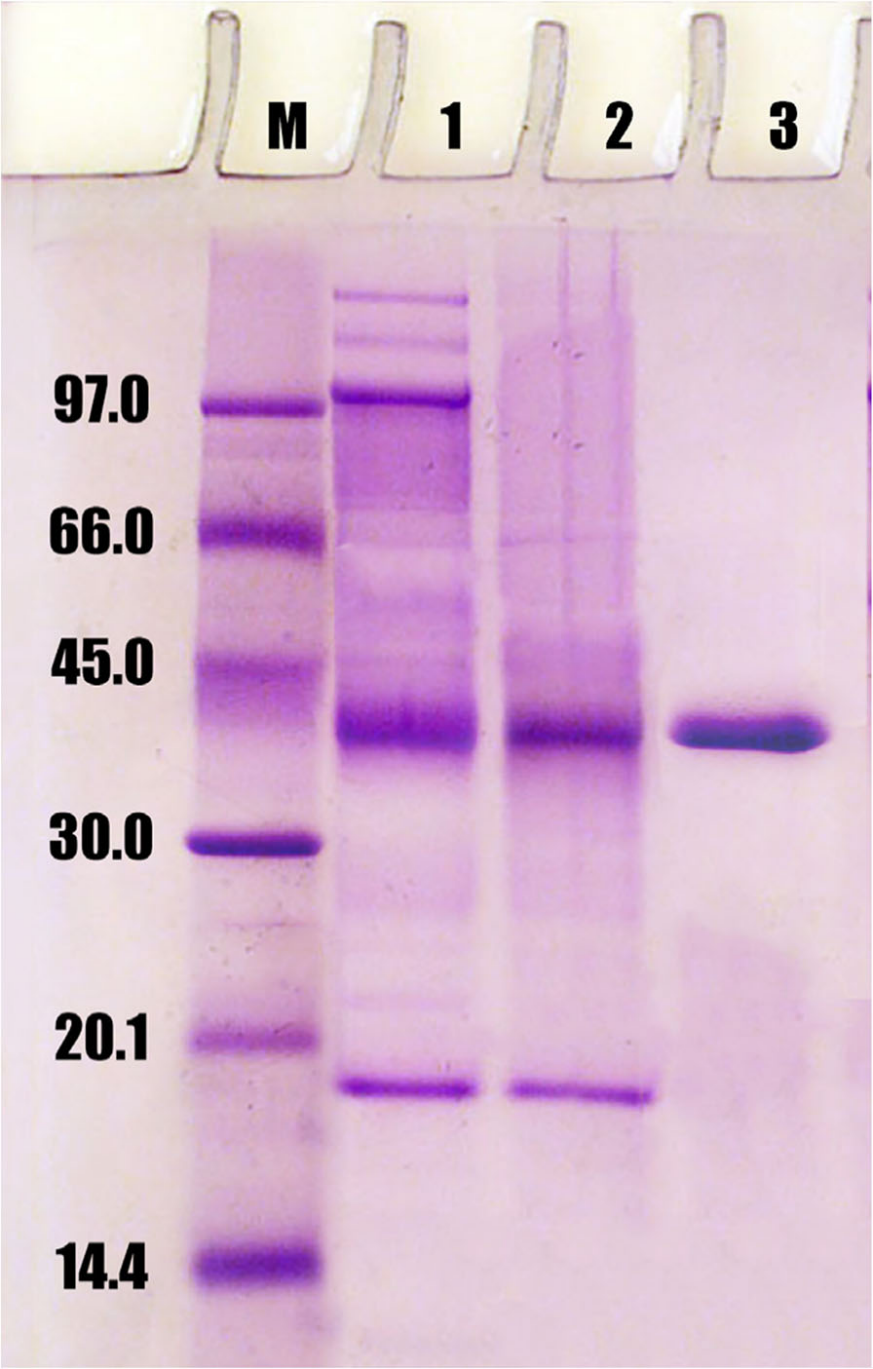
SDS-PAGE for miswak protease P1. 1, molecular markers, 2, crude extract, 3, DEAE-Sepharose P1, 4, Sephacryl S-200 P1

Two chromatography columns were used for purification of protease from miswak. By DEAE-Sepharose column, three isoforms of proteases (P1, P2 and P3) were eluted by gradient steps of NaCl at 0.0, 0.1 and 0.2 M, respectively (Fig. 1). Protease P1 possessed the most activity (412 units) and the highest specific activity (89.5 units/mg protein) compared with the other isoforms (Table 1). Protease P1 was chromatographed on Sephacry S-200 column (Fig. 2), where the specific activity and fold purification raised to 355 units/mg protein and 9.1 fold, respectively. The homogeneity of protease P1 was detected by SDS-PAGE (Fig. 3). The molecular mass of protease P1 was found to be 42 kDa by using Sephacryl S-200 and SDS-PAGE.

**Table 1:** Purification scheme for meswak protease

Steps	T. units	T. Protein mg	S.A^a^	Fold purification	Recovery 100%
Crude extract	545	14	39	1	100
Chromatography on DEAE-Sepharose					
0.0 M NaCl (P1)	412	4.6	89.5	2.29	75
0.1 M NaCl (P2)	40	2.2	18	0.46	7.3
0.2 M NaCl (P3)	60	6.5	9.2	0.23	11
Sephacryl S-200 P1	355	1.0	355	9.1	65

^a^*S.A* Specific activity (units/mg protein)

**Fig. 4:**
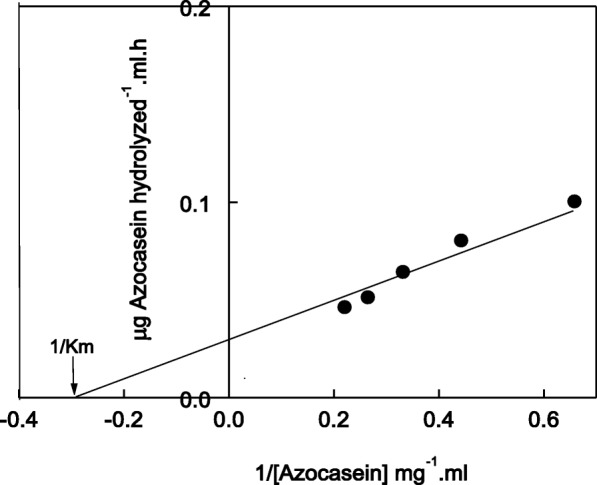
Reciprocal of Lineweaver-Burk plot relating miswak protease P1 reaction velocities to azocasein concentrations (1.5–4.5 mg). Km was calculated as mg azocasein/ml

The substrate specificity of protease P1 was detected by using different proteins (Table 2). The protease P1 digested proteins in the order of caseine > haemoglobin > egg albumin > gelatin > bovine serum albumin with 100, 95, 72, 68 and 53% residual activity, respectively. The kinetic of protease P1was detected by determining its Km. The Km of protease P1 was found to be 3.3 mg azocasein/ml by using reciprocal of Lineweaver-Burk plot (Fig. 4).

**Table 2:** Substrate specificity of miswak protease P1. Each value represents the mean of three experiments ± S.E

Substrate	Units/mg protein	Relative activity %
Casein	370 ± 18.5	100 ± 5.0
haemoglobin	350 ± 16.9	95 ± 4.6
Egg albumin	264 ± 13.9	72 ± 3.8
Gelatin	348 ± 20.4	68 ± 4.0
Bovine serum albumin	197 ± 10.4	53 ± 2.8

**Fig. 5:**
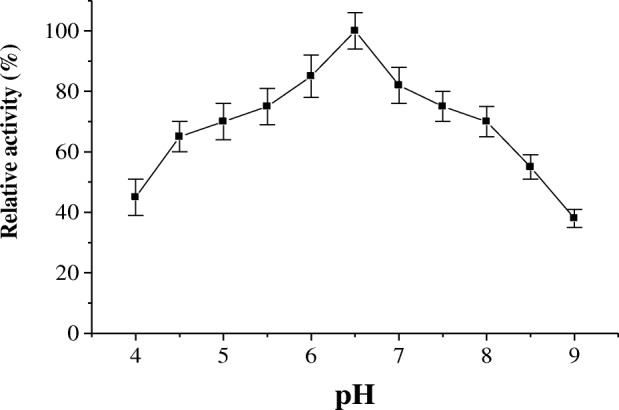
pH optimum of miswak protease P1. The reaction mixture contained in 1.0 ml: 3% azocasein, 100 μl of enzyme and 50 mM sodium acetate buffer (pH 4.0–6.5), and 50 mM Tris-HCl buffer (pH 7.0–9.0) and adjusted to 1 ml with distilled water. Each point represents the mean of three experiments ± S.E

**Fig. 6:**
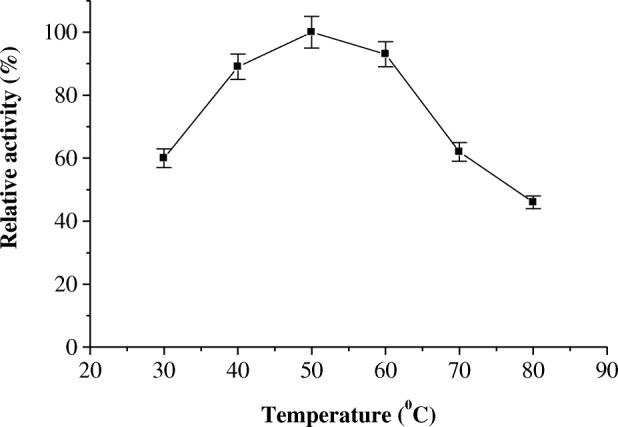
Temperature optimum of miswak protease P1. The enzyme activity was measured at various temperatures (30–80 °C) using the standard assay method. Each point represents the mean of three experiments ± S.E

**Fig. 7:**
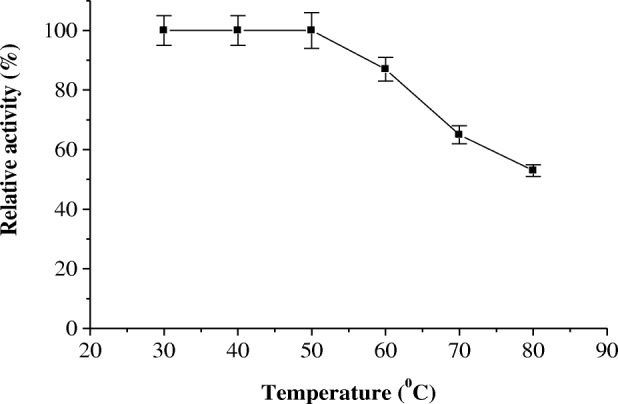
Effect of temperature on the thermal stability of miswak protease P1. The reaction mixture contained in 1.0 ml: 3% azocasein, 100 μl of enzyme and 50 mM sodium acetate buffer 6.5 and adjusted to 1 ml with distilled water. The reaction mixture was preincubated at various temperatures for 15 min prior to substrate addition, followed by cooling in an ice bath. The enzyme activity was measured using the standard assay method. Activity at zero time was taken as 100% activity. Each point represents the mean of three experiments ± S.E

The effect of pH on the activity of protease P1 was determined (Fig. 5). The pH optimum of protease P1 was detected at pH 6.5. The enzyme acts on acidic and alkaline sides of pH profile, where its residual activity % was 45 and 38 at pH 4 and 9, respectively. The temperature optimum of the protease P1 was determined from temperature profile (Fig. 6). The protease P1 had temperature optimum at 50 °C. The enzyme retained 40% of its activity at 80 °C. In the same manner, the protease P1was thermal stable up to 50 °C and the enzyme lost 50% of its activity at 80 °C after incubation for 1 h (Fig. 7).

The effect of metal ions (Ca^2+^, Ni^2+^, Pb^2+^, Co^2+^, Cu^2+^ and Zn^2+^) on the activity of the protease P1 was detected (Table 3). All metal ions caused partial inhibitory effect on the protease P1 except of Hg^2+^ which caused strong inhibitory effect. The study of protease inhibitors on the activity protease P1 was evaluated (Table 4). PMSF, 1,10 phenanthroline and EDTA caused slightly inhibitory effect on the activity protease P1, while *p*-HMB and iodoacetamide caused strong inhibitory effect.

**Table 3:** Influence of metal ions at 5 mM on miswak protease P1. Each value represents the mean of three experiments ± S.E

Metal cations	Units/mg protein	Relative activity %
Control	355 ± 12.4	100 ± 3.5
Ca^2+^	286 ± 15.0	80 ± 4.2
Ni^2+^	293 ± 13.5	82 ± 3.8
Pb^2+^	347 ± 16.4	97 ± 4.6
Co^2+^	286 ± 11.7	80 ± 3.3
Hg^2+^	164 ± 9.2	46 ± 2.6
Cu^2+^	268 ± 14.6	75 ± 4.1
Zn^2+^	272 ± 12.8	76 ± 3.6

**Table 4:** Effect of protease inhibitors at 2 mM on miswak protease P1. Each value represents the mean of three experiments ± S.E

Substrate	Units/mg protein	Relative activity %
Control	321 ± 17.3	100 ± 5.4
PMSF	289 ± 13.4	90 ± 4.2
1,10 Phenathroline	304 ± 13.1	95 ± 4.1
EDTA	293 ± 12.5	91 ± 3.9
p-HMB	71 ± 3.8	22 ± 1.2
Iodoacetamide	118 ± 5.1	35 ± 1.5

## Discussion

In this study, protease was purified and characterized from miswak roots. After two steps of chromatography, the homogeneity of protease P1 was detected by SDS-PAGE. The molecular mass of protease P1 was found to be 42 kDa. Different molecular weights (30–45 kDa) were detected for cysteine proteases from horse gram [28], *Curcuma longa* [29], *Euphorbia nivulia* [30] and ginger rhizome [31].

The substrate specificity of protease P1 showed that the enzyme acted on caseine, haemoglobin, egg albumin, gelatin and bovine serum albumin. Similar digestion was detected for horse gram cysteine protease toward the substrates [28]. The Km of protease P1 was found to be 3.3 mg azocasein/ml. Similar Km (2.8 mg azocasein/ml) was detected in germinated wheat cysteine protease [32]. The high value of Km (6.74 mg azocasein/ml) was detected for onion [33].

The maximum activity of protease P1 was detected at pH 6.5. However, horse gram cysteine protease showed higher activity at pH 5.5 [28]. The acidic pH optimum of germinated wheat cysteine protease was detected at 4.0 [32]. The protease P1 had temperature optimum at 50 °C and thermal stability up to 50 °C. The maximum activity of cysteine proteases from horse gram and *Euphorbia microsciadia* was detected at 40 °C and 45 °C, respectively [3, 28].

All metal examined caused partial inhibitory effect on the protease P1. However, the inhibitory effect of Hg^2+^ was strong indicated that the protease P1 is cystein protease. This agreed with cysteine proteases from *Euphorbia microsciadia* and horse gram which inhibited by Hg^2+^ [3, 28]. The inhibition of the activity of protease P1 by protease inhibitors determined the types of protease. The slightly inhibitory effect of PMSF, 1,10 phenanthroline and EDTA on the activity of protease P1 showed that the enzyme did not serine or metaloprotease. *p*-HMB and iodoacetamide caused strong inhibitory effect on the activity of the protease P1 indicating the enzyme is cysteine protease. Similar inhibitory effects were detected for cysteine proteases from *Euphorbia microsciadia* [3], horse gram [28] and ginger rhizome [31].

From the above findings the miswak protease P1, as cysteine protease, could be used in toothpaste for oral hygiene. However, Pleszczyńska et al. [34] studied the potential applications of enzymes in the treatment and prevention of oral diseases. Proteases of plant origin have been tested for removal of tooth stains and calculus [35]. A papain, as cysteine protease, gel has been used for removal of caries, which eliminates infected dentin and simultaneously preserves a healthy dental structure [36].

## Conclusions

The study indicated that the purified miswak protease P1 is cysteine protease depending on the study of the inhibition by cysteine protease inhibitors and Hg^2+^. Depending on cysteine proteases such as papain are used in oral gel, miswak protease P1may be digested the protein residues in the oral when the miswak is used as natural toothbrush. The biochemical characterization of this enzyme will be display the suitable conditions for using this enzyme in toothpaste in the future and the enzyme may be used in other applications.

## Abbreviations

EDTA: Ethylenediaminetetraacetic acid
*p*-HMB: *p*-Hydroximercuribenzaoic acid
PMSF: Phenylmethylsulfonyl fluoride
SDS-PAGE: Sodium dodecyl sulphate
